# A three-country comparison of psychotropic medication prevalence in youth

**DOI:** 10.1186/1753-2000-2-26

**Published:** 2008-09-25

**Authors:** Julie M Zito, Daniel J Safer, Lolkje TW de Jong-van den Berg, Katrin Janhsen, Joerg M Fegert, James F Gardner, Gerd Glaeske, Satish C Valluri

**Affiliations:** 1 Pharmaceutical Health Services Research, School of Pharmacy, University of Maryland, Baltimore, Maryland, USA; 2 Department of Psychiatry, School of Medicine, University of Maryland, Baltimore, Maryland, USA; 3 Departments of Psychiatry and Pediatrics, Johns Hopkins Medical Institutions, Baltimore, Maryland, USA; 4 Department of Social Pharmacy, Pharmacoepidemiology & Pharmacotherapy, Groningen University for Drug Exploration (GUIDE), Groningen, The Netherlands; 5 Arzneimittelepidemiologie und Public Health, University of Bremen, Bremen, Germany; 6 Department of Child and Adolescent Psychiatry/Psychotherapy, University Hospital Ulm, Germany

## Abstract

**Background:**

The study aims to compare cross-national prevalence of psychotropic medication use in youth.

**Methods:**

A population-based analysis of psychotropic medication use based on administrative claims data for the year 2000 was undertaken for insured enrollees from 3 countries in relation to age group (0–4, 5–9, 10–14, and 15–19), gender, drug subclass pattern and concomitant use. The data include insured youth aged 0–19 in the year 2000 from the Netherlands (n = 110,944), Germany (n = 356,520) and the United States (n = 127,157).

**Results:**

The annual prevalence of any psychotropic medication in youth was significantly greater in the US (6.7%) than in the Netherlands (2.9%) and in Germany (2.0%). Antidepressant and stimulant prevalence were 3 or more times greater in the US than in the Netherlands and Germany, while antipsychotic prevalence was 1.5–2.2 times greater. The atypical antipsychotic subclass represented only 5% of antipsychotic use in Germany, but 48% in the Netherlands and 66% in the US. The less commonly used drugs e.g. alpha agonists, lithium and antiparkinsonian agents generally followed the ranking of US>Dutch>German youth with very rare (less than 0.05%) use in Dutch and German youth. Though rarely used, anxiolytics were twice as common in Dutch as in US and German youth. Prescription hypnotics were half as common as anxiolytics in Dutch and US youth and were very uncommon in German youth. Concomitant drug use applied to 19.2% of US youth which was more than double the Dutch use and three times that of German youth.

**Conclusion:**

Prominent differences in psychotropic medication treatment patterns exist between youth in the US and Western Europe and within Western Europe. Differences in policies regarding direct to consumer drug advertising, government regulatory restrictions, reimbursement policies, diagnostic classification systems, and cultural beliefs regarding the role of medication for emotional and behavioral treatment are likely to account for these differences.

## Background

Increased psychotropic medication prevalence for youth has been reported during the last decade in the UK, Germany, Italy, Denmark, and the Netherlands, as well as in the US. Drug subclasses that have increased the most have been the selective serotonin reuptake inhibitor (SSRI) antidepressants and the atypical antipsychotics [1-4]. There are, nonetheless, major cross-national differences in psychotropic prevalence by drug class and subclass, gender and age group [5].

The variability in US-European psychotropic medication practice patterns reflects many differences such as diagnostic systems, practice guidelines, drug regulations, decentralized private vs. centralized national health service delivery systems, availability and financing of services as well as cultural beliefs [6].

Social attitudes and regulatory restrictions have been suggested as contributing factors [6-8]. Countries such as Germany, France and Italy have major government restrictions–in part due to the high costs of newer psychotherapeutic drugs and concerns about stimulant misuse. Government reimbursement of services is more ample in Europe. Nevertheless, US-European variations are well studied regarding the extent of referrals to specialists [9], test ordering [10], clinical preferences for the treatment of coronary heart disease [11], common surgical procedures [12], and Caesarean section birth deliveries [13]. With respect to the sociology of medicine, each country may imprint its own particular culture; in the US this reflects its individualist and activist therapeutic mentality [14].

### Aims of the study

The study aims to compare psychotropic drug use cross-nationally among 3 Western countries. The outcome data presented for the year 2000 were the prevalence of stimulant, antipsychotic and antidepressant medication and any psychotropic use in youth aged 0–19 years from 2 major European countries and the US. Drug class data from each country were compared with respect to total prevalence and were stratified by age and gender.

## Methods

Administrative claims data for youth aged 0–19 years enrolled in selected large health insurance systems in the Netherlands, Germany and the US were examined for the year 2000. Claims records were organized with patient as the unit of analysis and duplicate records were removed. The treatment data were restricted to youth in outpatient settings.

### Data sources

#### Netherlands data

were derived from pharmacy dispensing files from the Inter-Action database (IADB.nl). The IADB comprises all prescriptions from approximately 400,000 people in north-eastern Netherlands. This database includes all prescriptions regardless of prescribing specialty, insurance, or reimbursement status, apart from OTC drugs. Youth aged 0 through 19 numbered 110,944 during 2000.

#### German data

were derived from individual level prescription data from the Gmuender ErsatzKasse (GEK), one of about 270 different statutory health insurance companies in Germany. Nearly 90% of the 82 million German inhabitants are members of a statutory health insurance company. Although many such companies are quite small and represent only regional participation, the GEK comprises 1.6 million members located in all regions of Germany. The data from the GEK are representative of the 72 million Germans who are enrolled in a statutory health insurance company (SHIC). The data file for this analysis comprised 356,520 enrollees who were less than 20 years old in 2000.

#### United States data

were derived from administrative claims files from a narrowly defined population of youth whose family income (upper limit is twice the federal poverty limit) qualified them for inclusion in the state-Children's Health Insurance Program (s-CHIP) of a mid-Atlantic state. This population is similar to US privately insured children in terms of age distribution, race and family composition but moderately lower in parental education and employment. Nevertheless, s-CHIP and privately insured children are largely similar in health status [15]. During the year 2000, s-CHIP comprised 127,157 youth. Both prescription files and enrollment data were used in the analysis.

### Measures

Annual prevalence was defined as the dispensing of 1 or more prescriptions for a psychotropic drug during the study year (2000) per 100 enrolled youth. Prevalence was stratified by age and gender. Nine classes of psychotropic drugs included: antidepressants, antipsychotics, alpha-agonists, anxiolytics, hypnotics, lithium, antiparkinsonian agents, anticonvulsant-mood stabilizers and stimulants. Antidepressant subclasses included selective serotonin reuptake inhibitors (SSRI), tricyclic antidepressants (TCA) and other antidepressants. Antipsychotic subclasses included atypical and conventional antipsychotics. Stimulants included methylphenidate and amphetamine products. Anticonvulsant-mood stabilizers (ATC-MS) included carbamazepine, divalproex/valproic acid, lamotrigine, gabapentin and topiramate. Cross-national comparisons of any psychotropic medication use presents a challenge, in that anticonvulsant-mood stabilizers are used far more commonly in the US for psychiatric purposes than in Europe. Unfortunately, the study data did not have diagnoses available on indications for their use. Consequently, to improve the validity of anticonvulsants for mood stabilizer use, we restricted the analysis to ATC-MS users who additionally had one or more psychotropic classes in the study year, thereby excluding those most likely to receive these medications for the treatment of seizure disorder. Concomitant drug use refers to combinations of medications used concurrently and the analysis compared monthly combination drug dispensing within 3 time frames: 1 year, 3 months and 1 month, to assess the effect of each time frame on the prevalence of co-prescription. As in the prevalence of any psychotropic medication use, concomitant use with ATC-MS data was adjusted by excluding individuals who had ATC-MS dispensed but no other psychotropic medications during the study year.

### Analysis

The cross-sectional analysis describes the total, age-and gender-specific prevalence across three countries. The age and gender distributions of the enrolled youth (denominator) were adjusted applying the direct standardization method and using the 2000 US census population estimates as the standard population [16]. This adjustment corrects for the imbalanced age distribution caused by the US data with its higher proportion of 0–4 year olds and permits fair comparison across countries. Annual prevalence and the 95% confidence intervals (Cls) estimated by the exact method [17] are presented. Confidence intervals at the 95% level for these standardized total estimates were obtained by the Chiang method [18]. Prevalence ratios were calculated to compare countries and the 95% CIs for ratios were based on the method of Dawson and Trapp [19]. The frequency of concomitant use was calculated and the highest ranking combinations were assessed for each country.

## Results

### Prevalence findings

Table 1 presents the study population of youth from the US, the Netherlands, and Germany by age group and gender. The total number of enrollees in 2000 was 127,157 (US), 110,994 (Netherlands) and 356,520 (Germany). Youth 0–4 years of age represented 51.7% of the US enrollees, 24.7% of the Dutch and 21.0% of the German enrollees. To address this disparity, prevalence data were adjusted to the distribution by age group of youths in the US 2000 census.

**Table 1:** Age and gender characteristics for enrolled youth in 3 countries during 2000

	US			Netherlands			Germany		
Age (yr)	Male	Female	Total	Male	Female	Total	Male	Female	Total

0–4	33,419	32,316	65,735	14,069	13,295	27,364	38,473	36,774	75,247
5–9	13,016	12,492	25,508	13,296	12,806	26,102	45,236	43,055	88,291
10–14	9,828	9,601	19,429	13,246	13,140	26,386	52,185	49,710	101,895
15–19	7,117	9,374	16,485	15,580	15,512	31,092	46,784	44,303	91,087

Total	63,374	63,783	127,157	56,191	54,753	110,944	182,678	173,842	356,520

Data in Table 2 show the rank order of annual prevalence use of any psychotropic by country as 6.7% (US), 2.9% (Netherlands), and 2.0% (Germany). The prevalence differences are reflected in the prevalence ratio analyses which show that US usage was 2.27 (CI = 2.22, 2.32) and 3.33 (CI = 3.27, 3.40) times more likely than Dutch and German usage, respectively. Dutch usage was significantly greater than German usage [prevalence ratio of 1.47 (CI = 1.44, 1.51)]. The one year prevalence of receiving one or more of any psychotropic during 2000 was highest in all countries at ages 10–14 years for males and ages 15–19 for females. German youth led the 0–4 year-old rank order of prevalence of any psychotropic (1.63%), while Netherlands and US rates were equivalent (0.9%).

**Table 2:** Prevalence per 100 and 95% CIs for the use of any psychotropic drug during the year 2000

	US (n = 127,157)			Netherlands (n = 110,944)			Germany (n = 356,520)		
Age(yr)	Male	Female	Total*	Male	Female	Total*	Male	Female	Total*

0–4	1.21	0.52	0.88	1.00	0.71	0.86	1.86	1.38	1.63
	1.10–1.34	0.45–0.61	0.87–0.88	0.84–1.18	0.58–0.87	0.85–0.87	1.73–2.00	1.26–1.51	1.62–1.63
5–9	11.95	4.38	8.25	3.99	1.30	2.68	2.85	1.19	2.04
	11.39–12.52	4.03–4.75	8.25–8.26	3.66–4.33	1.11–1.52	2.67–2.69	2.69–3.00	1.09–1.30	2.04–2.04
10–14	14.16	5.97	10.17	5.38	1.95	3.71	3.37	1.33	2.38
	13.48–14.87	5.5–6.46	10.16–10.18	5.00–5.78	1.72–2.2	3.70–3.72	3.22–3.53	1.23–1.44	2.37–2.38
15–19	7.62	6.30	6.98	4.35	4.44	4.40	1.75	2.12	1.93
	7.01–8.26	5.82–6.82	6.97–6.99	4.04–4.68	4.12–4.78	4.39–4.40	1.63–1.87	1.99–2.26	1.93–1.93

Total*	8.87	4.35	6.66	3.72	2.11	2.94	2.47	1.50	2.00
	8.86–8.87	4.34–4.35	6.66–6.67	3.72–3.73	2.11–2.12	2.94–2.94	2.47–2.47	1.5–1.51	2.00–2.00

*Totals were adjusted to the child and adolescent population of the US 2000 census by the direct standardization method.

Table 3 illustrates that there was a limited but disparate use of lithium (< .01% in German, 0.01% in Dutch and 0.15% in US youth) and antiparkinsonian agents (0.01% in German and Dutch and 0.05% in US youth). Anxiolytic use was greater in Dutch youth than in German and US youth, respectively: 0.73% compared to 0.41% and 0.49%. Hypnotic use was twice as common in Dutch youth compared with US but scarcely used in German youth (0.09%). There was a wide disparity across countries in alpha-agonist use which was 9-fold and 120-fold more common in US youth than in Dutch and German youth, respectively.

**Table 3:** Prevalence per 100 and 95% CIs for the use of six* selected psychotropic drugs during the year 2000

	US (n = 127,157)			Netherlands (n = 110,944)			Germany (n = 356,520)		
	Male	Female	Total*	Male	Female	Total*	Male	Female	Total*

Alpha-Agonist	0.74	0.18	0.47	0.07	0.02	0.05	0	0	0
	0.62–0.86	0.14–0.22	0.43–0.51	0.05–0.1	0.01–0.03	0.03–0.07	0–0.01	0–0.3	0–0.03
Lithium	0.18	0.13	0.15	0	0.01	0.01	0	0	0
	0.08–0.25	0.06–0.21	0.07–0.23	0–0.02	0–0.02	0–0.02	0–0	0–0.01	0–0.1
Anxiolytic	0.51	0.47	0.49	0.65	0.81	0.73	0.4	0.42	0.41
	0.46–0.58	0.41–0.54	0.42–0.55	0.6–0.74	0.74–0.92	0.68–0.81	0.36–0.44	0.38–0.46	0.38–0.44
Hypnotic	0.15	0.17	0.16	0.35	0.32	0.33	0.08	0.11	0.09
	0.12–0.2	0.14–0.21	0.14–0.21	0.31–0.41	0.27–0.4	0.3–0.39	0.07–0.09	0.1–0.14	0.07–0.13
Antiparkinsonian	0.07	0.04	0.05	0.01	0.01	0.01	0.01	0.01	0.01
	0.03–0.09	0.01–0.07	0.02–0.07	0–0.02	0–0.02	0.01–0.02	0.01–0.02	0–0.02	0.0–0.02
ATC-MS	1.03	0.49	0.77	0.36	0.38	0.37	0.39	0.37	0.38
	0.94–1.12	0.42–0.54	0.72–0.84	0.32–0.42	0.32–0.43	0.33–0.41	0.37–0.43	0.35–0.41	0.37–0.41

*Of the 9 classes comprising “any psychotropic prevalence”. Data on antipsychotics, stimulants and antidepressants are shown in Tables 4, 5 and 6, respectively.

*Totals were adjusted to the child and adolescent population of the US 2000 census by the direct standardization method.

Antipsychotic prevalence in the countries assessed for year 2000 is presented on Table 4. In rank order, the prevalence of antipsychotics was 0.76% (US), 0.51% (Netherlands), and 0.34% (Germany). Though the total antipsychotic cross-national prevalence differences were relatively modest, Germany's prevalence was strikingly different in three respects. Atypical antipsychotics represented only 5% of the total in Germany, but 48% in the Netherlands and 66% in the US. The antipsychotic gender ratio (M:F) was distinctly lower in Germany (1.4:1) compared to the Netherlands (3.2:1) and the US (2.8:1). Furthermore, among 0–4 year olds, German youth had the highest antipsychotic prevalence (0.64%), followed by the Netherlands (0.10%), and the US (0.07%), a stark reversal of the leading usage trend observed in other drug classes, e.g. antidepressants and stimulants.

**Table 4:** Prevalence per 100 and 95% CIs for the use of antipsychotics during the year 2000

	US (n = 127,157)			Netherlands (n = 110,944)			Germany (n = 356,520)		
Age (yr)	Male	Female	Total*	Male	Female	Total*	Male	Female	Total*

0–4	0.11	0.02	0.07	0.14	0.05	0.10	0.74	0.53	0.64
	0.08–0.15	0.09–0.45	0.06–0.07	0.09–0.22	0.02–0.11	0.09–0.10	0.65–0.83	0.45–0.60	0.63–0.64
5–9	1.04	0.20	0.63	0.76	0.16	0.47	0.29	0.16	0.23
	0.87–1.23	0.13–0.30	0.62–0.64	0.62–0.92	0.10–0.24	0.46–0.47	0.24–0.34	0.12–0.20	0.22–0.23
10–14	1.57	0.56	1.08	1.26	0.29	0.79	0.27	0.14	0.21
	1.33–1.83	0.42–0.73	1.07–1.09	1.08–1.47	0.21–0.4	0.78–0.79	0.22–0.31	0.11–0.18	0.20–0.21
15–19	1.60	0.80	1.21	0.85	0.45	0.66	0.30	0.32	0.31
	1.32–1.92	0.63–1.00	1.20–1.22	0.71–1.00	0.35–0.57	0.65–0.66	0.26–0.36	0.27–0.38	0.31–0.31

Total*	1.10	0.40	0.76	0.76	0.24	0.51	0.39	0.28	0.34
	1.09–1.10	0.40–0.40	0.75–0.76	0.76–0.77	0.24–0.24	0.51–0.51	0.39–0.40	0.28–0.28	0.34–0.34

*Totals were adjusted to the child and adolescent population of the US 2000 census by the direct standardization method.

As shown in Table 5, the prevalence of stimulants for youth was 4.3% in the US, 1.2% in the Netherlands, and 0.7% in Germany. Stimulant prevalence peaked in all three countries at ages 10–14 years. In 0–4 year-olds, the US stimulant prevalence was 0.5%, 10–25 times higher than that of the two Western European countries. The stimulant gender ratio (M:F) in the US was 3.4:1, whereas it was 5.3:1 to 4.8:1 in Germany and the Netherlands. In the US, methylphenidate and amphetamine compounds were prescribed equivalently, whereas in the two Western European countries, over 95% of prescribed stimulant use was for methylphenidate.

**Table 5:** Prevalence per 100 and 95% CIs for the use of stimulants during the year 2000

	US (n = 127,157)			Netherlands (n = 110,944)			Germany (n = 356,520)		
Age (yr)	Male	Female	Total*	Male	Female	Total*	Male	Female	Total*

0–4	0.76	0.20	0.49	0.08	0.02	0.05	0.02	0.01	0.02
	0.67–0.86	0.15–0.25	0.48–0.49	0.04–0.14	0.00–0.05	0.04–0.06	0.01–0.04	0.00–0.03	0.01–0.02
5–9	10.72	3.68	7.29	2.86	0.63	1.77	1.74	0.40	1.09
	10.19–11.26	3.36–4.03	7.28–7.29	2.58–3.16	0.50–0.78	1.76–1.78	1.62–1.87	0.34–0.46	1.08–1.09
10–14	11.43	3.16	7.40	3.57	0.59	2.12	2.37	0.48	1.45
	10.80–12.07	2.82–3.53	7.39–7.41	3.26–3.9	0.46–0.73	2.11–2.12	2.24–2.50	0.42–0.55	1.45–1.45
15–19	2.75	0.59	1.70	1.17	0.22	0.71	0.42	0.06	0.25
	2.39–3.16	0.44–0.76	1.69–1.71	1.01–1.35	0.15–0.31	0.70–0.71	0.36–0.48	0.04–0.09	0.24–0.25

Total*	6.52	1.94	4.29	1.95	0.37	1.18	1.16	0.24	0.71
	6.52–6.53	1.94–1.95	4.29–4.29	1.95–1.96	0.37–0.37	1.18–1.18	1.16–1.16	0.24–0.24	0.71–0.71

*Totals were adjusted to the child and adolescent population of the US 2000 census by the direct standardization method.

Table 6 presents the antidepressant prevalence for youth cross-nationally. In rank order, the prevalence for 2000 was 2.7% (US), 0.5% (Netherlands), and 0.2% (Germany).

**Table 6:** Prevalence per 100 and 95% CIs for the use of antidepressants during the year 2000

	US (n = 127,157)			Netherlands (n = 110,944)			Germany (n = 356,520)		
Age (yr)	Male	Female	Total*	Male	Female	Total*	Male	Female	Total*

0–4	0.14	0.06	0.10	0.02	0.02	0.02	0.03	0.00	0.02
	0.10–0.19	0.04–0.09	0.10–0.10	0.00–0.06	0.01–0.07	0.02–0.02	0.01–0.05	0.00–0.01	0.01–0.02
5–9	2.24	0.74	1.51	0.30	0.09	0.20	0.13	0.09	0.11
	1.99–2.50	0.59–0.90	1.50–1.52	0.22–0.41	0.05–0.16	0.19–0.20	0.10–0.17	0.06–0.12	0.11–0.11
10–14	4.67	3.26	3.98	0.57	0.30	0.44	0.18	0.09	0.14
	4.26–5.11	2.91–3.64	3.97–3.99	0.45–0.71	0.22–0.41	0.43–0.44	0.14–0.22	0.07–0.12	0.13–0.14
15–19	5.03	5.21	5.12	1.16	1.74	1.44	0.29	0.58	0.43
	4.53–5.56	4.77–5.68	5.11–5.13	1.00–1.34	1.54–1.96	1.44–1.45	0.24–0.34	0.51–0.65	0.43–0.43

Total*	3.06	2.34	2.71	0.52	0.54	0.53	0.16	0.19	0.17
	3.06–3.07	2.34–2.34	2.71–2.71	0.52–0.52	0.54–0.54	0.53–0.53	0.16–0.16	0.19–0.19	0.17–0.18

*Totals were adjusted to the child and adolescent population of the US 2000 census by the direct standardization method.

In Germany and the Netherlands, 15–19 year olds were over 3 times more likely to utilize antidepressants than 10–14 year olds, whereas in the US the 15–19 year old group use was only 28% higher than in the younger aged group. In the US, only 14.8% of those on antidepressants were prescribed the TCA antidepressant subclass, whereas the proportion for TCAs was 48% in the Netherlands and 73% in Germany.

### Concomitant psychotropic patterns

To assess concomitant therapy in 3 time frames, 1-month (April 2000), 3-month (April through June) and 12-month time periods were used to measure the one-month co-occurrence of psychotropic classes for youth in the US dataset. There was a linear increase in co-occurring use as the time period widened: 19.2%, 23.9% and 27.0%. For the present study, the most conservative approach, (monthly co-occurrence) was adopted to avoid exaggerated estimates. Combinations were assessed from the following classes: stimulants, antidepressants, anxiolytics/hypnotics, alpha-agonists, antipsychotics, anticonvulsant-mood stabilizers and lithium. Of the 1908 medicated youth in the US group, concomitant therapy (defined as monthly co-occurrence) applied to 19.2% and ranged from pairs (n = 279), triplets (n = 80), quadruplets (n = 7) to 6 drug classes (n = 1). The leading pairs were stimulants with antidepressants (33.7%) and stimulants with alpha-agonists (18.3%). Dutch concomitant use was substantially less common: 8.5% had combined therapy almost entirely as pairs (77/80), of which stimulants and antipsychotics were the leading combination. German concomitant use affected only 5.9% of medicated youth and the use was entirely pairs except for one triplet. Since the bulk (62%) of the German combinations involved anticonvulsant-mood stabilizer and an anxiolytic/hypnotic, it is not possible to determine the extent of seizure disorder treatment. The other German pairs were ranked as follows: stimulant and antipsychotic (8.9%), anticonvulsant-mood stabilizer and antipsychotic (7.6%) and stimulant and anticonvulsant-mood stabilizer (6.3%). Concomitant use with anticonvulsant-mood stabilizers affected 5.8% (110/1908) of US medicated youth, 1.9% (18/937) of medicated Dutch youth and 4.6% (62/1358) of medicated German youth.

## Discussion

The major finding of this cross-national prevalence study of psychotropic medications prescribed for youth is that the US prevalence exceeds Western European prevalence for overall psychotropic use and that drug class rates differ cross-nationally. While US stimulant and antidepressant use far exceeded the rates in Western Europe, the rates between the countries for antipsychotic use were less disparate. Findings from published studies from various Western European countries generally match the prevalence reports for the 3 major psychotropic classes (stimulants, antidepressants and antipsychotics) in Germany and the Netherlands as detailed below.

### Broad cross-national trends

In a review of 10 Medline reports of published studies of prevalence of psychotropic medications prescribed for youth in Western European countries during the period from 1999 to 2002, there was general agreement on their low rates of use of psychotropic medications in youth relative to published reports of US utilization [4,20-29]. Stimulant prevalence was particularly low in France (0.05%) [20], but relatively higher (1.0%) in the Netherlands. Consistent with previous findings, antidepressant use is more common in the US. In a four-country antidepressant analysis, use of more than one antidepressant during the year 2000 was approximately four times more frequent in US youth (21.3%) than in Dutch (5.9%), German (5.4%), and Danish (5.6%) youth [27]. The striking antidepressant subclass pattern of the present study shows SSRIs represent nearly two-thirds of antidepressant use in US and Dutch youth, but less than one-quarter of German antidepressant use. The prevalence of antipsychotics in youth aged 0–4 ranged from 0.13% in Italy [24] to 0.5% in the Netherlands [29]. Generally, these antipsychotic prevalence findings closely matched those of this study, indicating that US youth -compared to Western Europeans-have a far higher prevalence of stimulants and antidepressants, but a less disparate prevalence of antipsychotics. Patterns for less commonly used psychotropic medications were remarkably similar across the 3 countries for lithium, alpha-agonists and antiparkinsonian agents but Dutch usage led the other countries in anxiolytic and hypnotic use. In the following sections, several factors that influence the utilization of psychotropic drugs across countries are presented.

### Regulatory differences

Amphetamines are seldom prescribed in Western Europe. In fact, they were not allowed to be prescribed in France [20,30], Spain [31], and Italy [30], at the time of this study. Government cost restrictions in Europe have also cut down on the use of expensive drugs, particularly with respect to patent-protected antipsychotics and antidepressants [1,32]. These year 2000 patterns may be expected to change as recent European data suggest [30].

### Diagnostic classification differences

The International Classification of Diseases (ICD-10) is now generally used for diagnostic purposes in Western Europe. This fact can influence the frequency of diagnosis and through that to treatment. For example, the diagnosis of hyperkinetic disorder in the ICD is more stringent than that of attention deficit hyperactivity disorder (ADHD) in the US based on the Diagnostic and Statistical Manual (DSM) criteria [33,34]. However, there is evidence that conduct disorder is more readily diagnosed in the UK using the ICD than in the US with the DSM [35]. The US trend of increasing bipolar diagnosis in children and adolescents [36] does not reflect European practice [37].

### Drug class preferences

The common use of phenothiazine products in German youth aged 0–4 may be due to its medical usage for antihistaminic effects or to induce sleep, and not for psychiatric indications. In the US, several phenothiazines, e.g. promethazine, have antihistaminic properties which have been used to treat allergy and cold symptoms, but these drugs are classified separately and were not assessed as psychotropic uses. That may not be the case in Europe. Similarly, in Sweden during the late 1970s and early 1980s, 10% of youth had received prescriptions for neuroleptic drugs before their 5^th ^birthday for sedative/hypnotic use [38]. The use of antidepressants varies by physician specialty depending on the setting and type of insurance. In year 2000, the prevalence of prescribed stimulant medication for 0–4 year-olds in Western Europe was quite low [UK (0%), Germany (0.02%), Netherlands (0.05%)] in relation to the US (0.49%) [39].

### Co-medication patterns

Use of multiple medications, i.e., having two or more prescribed psychotropic medications during a one year period, was rare in the Netherlands in 1999 compared to the US [21]. In the current study, US concomitant use was 2 or 3 times more common than in Dutch and German youth, respectively.

### Access to physician specialties

General practitioners prescribe most of the psychotropic drugs in Western Europe. In the US, pediatricians prescribe most of the stimulants for youth [40], whereas psychiatrists prescribe most of the antipsychotics [41]. In France, the first prescription of a stimulant must be written by a specialist. The general practitioner can continue stimulant prescribing, but only for a maximum period of one year [20]. The number of child psychiatrists per capita in Western Europe is low compared to the rate in the US [35], which presumably also accounts for some prescribing differences.

### Limitations

Several limitations should be noted: 1) These data are cross-sectional in nature, covering one year, which do not permit time trend analyses. Future studies should address changing patterns over time. 2) Diagnostic information was not available so that it is unclear if antidepressants were prescribed for depression, anxiety, obsessive compulsive disorder or other indications. 3) US direct-to-consumer prescription drug advertising and professional journal advertising may contribute to increased awareness and utilization of medication to treat emotional and behavioral conditions in children. 4) There is no information on reimbursement patterns. 5) Access to medical specialists differs. 6) The US data were based on the s-CHIP Medicaid data from one state and have limitations as a representative US dataset, but adjustments were made to improve generalizability, e.g. prevalence of use rates were adjusted for the greater proportion of 0–4 year-olds in s-CHIP. 7) The analysis of the major psychotropic drug classes in this study did not include certain commonly used over the counter (OTC) drugs that are not generally recognized as important. Examples include St. John's Wort–used prominently in Germany for the treatment of depression [1] and the extensive use of anxiolytics and hypnotics for adolescents in many European regions [22].

## Conclusion

Prominent differences in psychotropic medication prevalence patterns for youth exist between the US and Western Europe and within Western Europe. Understanding these differences should help clarify and hopefully improve our understanding of the various influences on psychotropic drug treatment.

## Competing interests

The authors declare that they have no competing interests.

## Authors' contributions

LTWJ, KJ, GG and JMZ provided data and participated in the design and analysis of the study. JFG and SCV provided computerized data management and statistical analysis. JMZ and DJS drafted the manuscript. JMF and LTWJ provided critical review.
